# Case Report: Internal Carotid Artery Thrombosis: A Rare Complication After Fibrin Glue Injection for Cavernous Sinus Hemostasis

**DOI:** 10.3389/fsurg.2021.730408

**Published:** 2021-11-02

**Authors:** Hugo Andrade-Barazarte, Zhongcan Chen, Chenyi Feng, Visish M. Srinivasan, Charuta G. Furey, Michael T. Lawton, Juha Hernesniemi

**Affiliations:** ^1^“Juha Hernesniemi” International Center of Neurosurgery, Cerebrovascular Diseases, Henan Provincial People's Hospital, Zhengzhou University, Zhengzhou, China; ^2^Department of Neurosurgery, St. Joseph's Hospital and Medical Center, Barrow Neurological Institute, Phoenix, AZ, United States

**Keywords:** anterior clinoidectomy, carotid artery thrombosis, cavernous sinus, fibrin glue injection, hemostasis

## Abstract

**Background:** Fibrin glue injection within the cavernous sinus (CS) is a demonstrably safe and simple technique to control venous bleeding with a low complication rate. However, this technique does have inherent risks. We illustrate 2 cases of internal carotid artery (ICA) thrombosis after fibrin glue injection in the CS for hemostasis.

**Methods:** After encountering this complication recently, we conducted a retrospective review of the surgical database of 2 senior neurosurgeons who specialize in cerebrovascular and skull base surgery to identify patients with any complications associated with the use of fibrin glue injection for hemostasis. Approval was given by respective institutional review boards, and patient consent was obtained.

**Results:** Of more than 10,000 microsurgery procedures performed by 2 senior neurosurgeons with a combined experience of 40 years, including procedures for aneurysms and skull base tumors, 2 cases were identified involving ICA thrombosis after fibrin glue injection in the CS for hemostasis. Both cases involved severe ischemic complications as a result of the ICA thrombosis. In this article, we present their clinical presentation, characteristics, management, and outcomes.

**Conclusion:** Direct injection of fibrin glue into the CS for hemostasis can effectively control venous bleeding and facilitate complex dissections. However, it can be associated with ICA thrombosis, with subsequent serious ischemia and poor prognosis. Although this complication appears to be rare, increased awareness of this problem should temper the routine use of fibrin glue in anterior clinoidectomy and transcavernous approaches.

## Introduction

Several neoplastic and vascular lesions located around the sellar and suprasellar regions require cavernous sinus (CS) exposure and anterior clinoidectomy (1–8). During these procedures, continuous venous bleeding from within and around the CS can obstruct surgical visibility and necessitate a blood transfusion. Therefore, a key step in such procedures is to achieve complete hemostasis in the opened CS before continuing with further surgery.

Techniques to obtain hemostasis during CS exposure include packing with hemostatic materials such as oxidized cellulose cotton or thrombin-soaked Gelfoam (Pfizer, Inc., New York, NY) and direct injection of fibrin glue into the bleeding points in the CS (6, 9–11). Fibrin glue, also called *fibrin sealant*, is injected directly into the Mullan (anteromedial) triangle or the Dolenc (clinoidal) triangle to provide effective hemostasis in the CS. Fibrin glue injection is associated with low morbidity, with only rare reports of temporary cranial nerve deficits or transient venous outflow changes (6, 10, 12, 13). More serious neurovascular complications of fibrin glue injections have not been reported in the neurosurgery literature. In this study, we searched the medical records database of 2 senior neurosurgeons specializing in cerebrovascular and skull base surgery, who have performed more than 10,000 microsurgery procedures for such conditions as aneurysms and skull base tumors, to identify any cases of internal carotid artery (ICA) thrombosis after fibrin glue injection in the CS for hemostasis.

## Case Identification

Our search of the medical records of more than 10,000 patients treated by the 2 neurosurgeons identified only 2 patients in whom ICA thrombosis caused by fibrin glue injection developed after a microsurgery procedure. This complication occurred in a 53-year-old man treated in 2018 for an unruptured aneurysm in the ophthalmic and clinoidal segment of the ICA and in a 64-year-old woman who underwent resection of a petroclival meningioma in 2019. We examine the clinical presentation, characteristics, management, and outcomes of these patients before and after diagnosing ICA thrombosis after fibrin glue injection. Approval was given by respective institutional review boards, and consent of the patients have been obtained.

## Clinical Presentation

### Case 1

**Figure 1:**
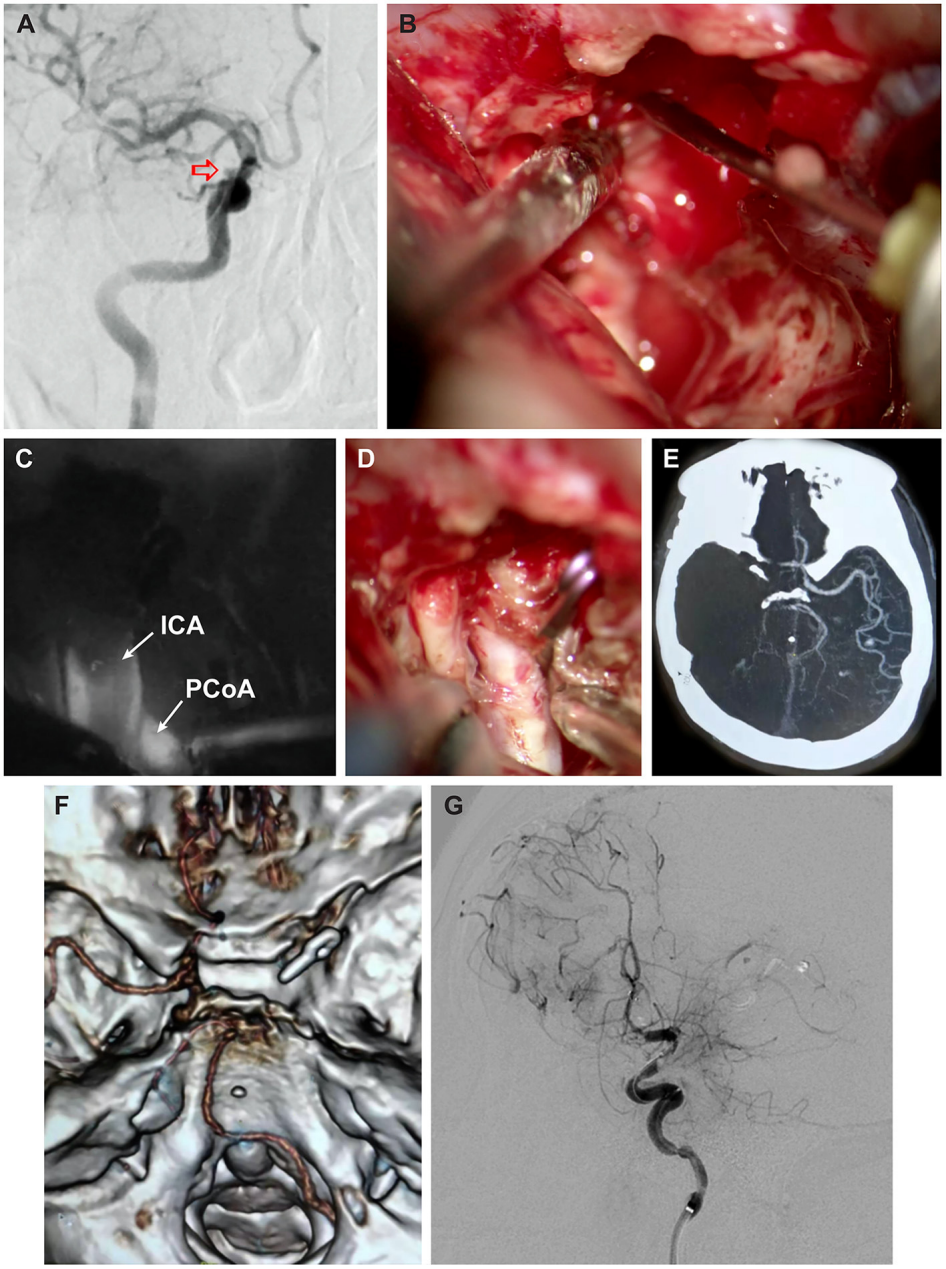
Case 1. **(A)** Digital subtraction angiography (DSA) oblique view demonstrating small unruptured aneurysm (*red arrow*) in the transition between the cavernous and clinoidal segments of the right internal carotid artery (ICA). **(B)** Intraoperative photograph of direct injection of fibrin glue into the cavernous sinus posterior to the ICA. **(C)** Indocyanine green (ICG) videoangiography demonstrating patency of the right ICA, darkened by the presence of atheroma in the center of the vessel, and patency of the right posterior communicating artery (PCoA) and **(D)** companion intraoperative photograph. **(E)** Postoperative axial computed tomography angiography (CTA) and **(F)** 3-dimensional CTA, superior skull base view, obtained 2 h after surgery, demonstrating complete occlusion of the right ICA. **(G)** Postoperative oblique-lateral DSA after several thrombectomies showing patency of the right ICA and contrast stagnation caused by poor collateral flow. Used with permission from Henan Provincial People's Hospital, Juha Hernesniemi International Center for Neurosurgery.

A 53-year-old man presented with intermittent right hemifacial spasm. Magnetic resonance angiography (MRA) demonstrated the absence of a vascular compression around the right facial nerve and the presence of a berry-like pouch in the ophthalmic segment of the ICA. Digital subtraction angiography (DSA) confirmed a small unruptured aneurysm located in the transition between the ophthalmic and clinoidal segments of the ICA (Figure 1A). Given the patient's positive medical history for hypertension and smoking, combined with his anxiety about a possible aneurysm rupture, he decided to undergo surgery. The patient underwent a right lateral supraorbital approach, as described elsewhere (14). After a tailored intradural anterior clinoidectomy and dissection of the distal dural ring were performed, continuous venous bleeding from the CS was controlled by injecting ~1 mL of fibrin glue (Baxter Healthcare Corp., Deerfield, IL), administered posterior to the clinoidal segment of the ICA (10). The bleeding persisted, requiring the injection of a second dose of fibrin glue at the same point (Figure 1B). The aneurysm was then clipped using a straight miniclip. Indocyanine green (ICG) videoangiography demonstrated patency of both the ICA and the posterior communicating artery (Figure 1C), with direct visualization noting stoppage of bleeding (Figure 1D). A piece of temporalis muscle soaked with fibrin glue was used to cover the defect around the anterior clinoidectomy and to prevent cerebrospinal fluid leakage. The procedure was uneventful, and the patient was transferred to the recovery room. However, ~2 h postoperatively, left-sided hemiplegia and somnolence developed, and emergent computed tomography (CT) (Figure 1E) and CTA (Figure 1F) demonstrated complete occlusion of the ICA. DSA showed thrombosis of the right ICA extending from its origin at the common artery to the right middle cerebral artery. The patient underwent several thrombectomies, which resulted in partial recanalization of the ICA. Hence, the presence of contrast stagnation distally on following angiograms (Figure 1G). A right-sided decompressive craniectomy was performed because of a dilated right pupil and CT showing complete right-sided ischemia. On postoperative day 4, the patient's clinical condition had further deteriorated (Glasgow Coma Scale 3 and both pupils dilated). CT demonstrated complete right-sided infarction, and the patient's family decided to withdraw care.

### Case 2

**Figure 2:**
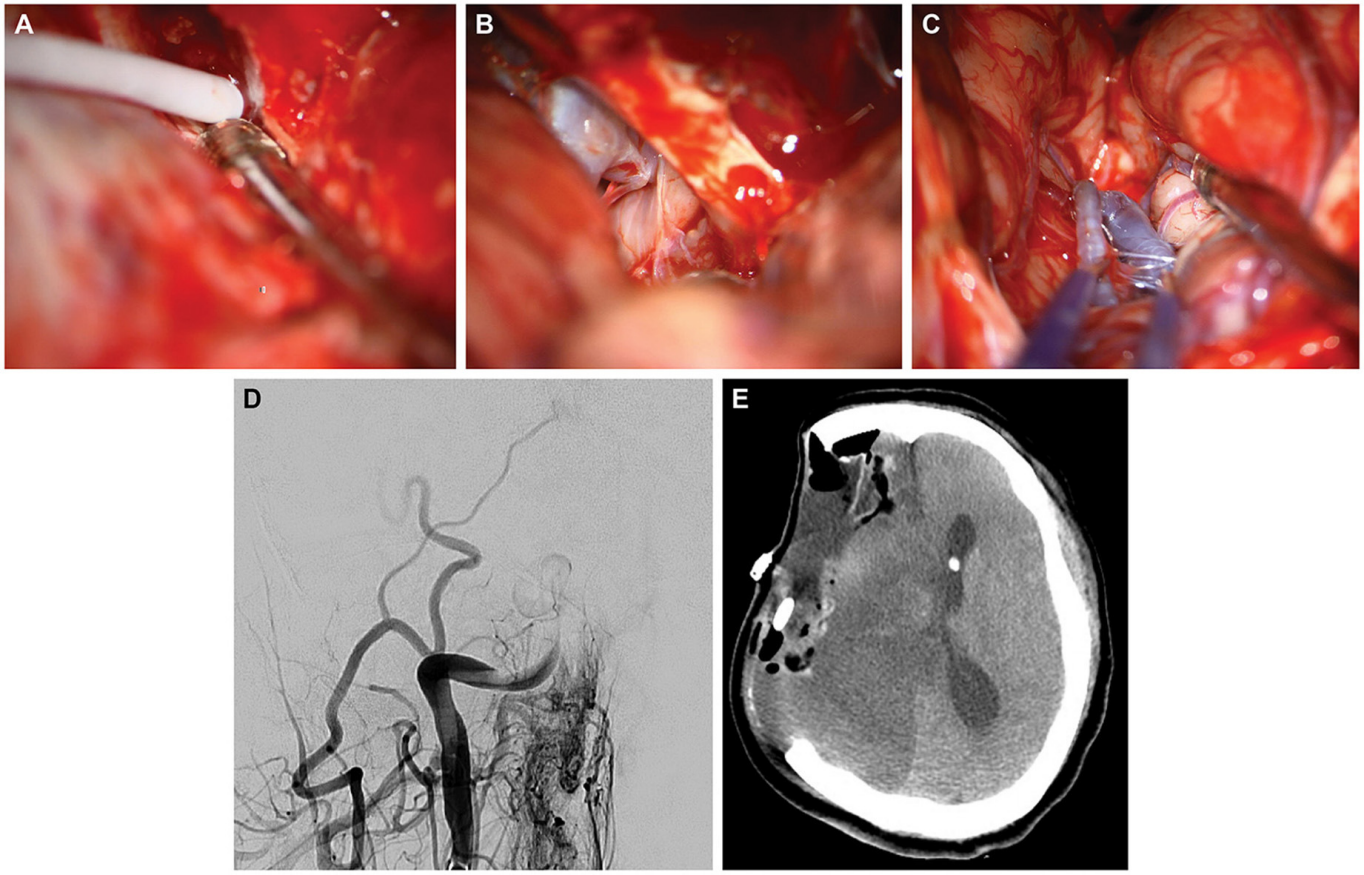
Case 2. Intraoperative photographs demonstrating **(A)** direct injection of fibrin glue into the cavernous sinus; **(B)** subsequent visualization of the patent internal carotid artery (ICA) during resection; and **(C)** 90 min later, the thrombosed middle cerebral artery, which has turned blue. **(D)** Postoperative posteroanterior angiography via the right common carotid artery demonstrating occlusion of the distal ICA. **(E)** Postoperative axial computed tomography of the head after decompressive hemicraniectomy and resection of ischemic territory. Used with permission from Barrow Neurological Institute, Phoenix, Arizona.

A 64-year-old woman presented with a petroclival meningioma that demonstrated continued growth. She underwent a right pterional craniotomy with a transcavernous approach and resection of the anterior clinoid process to resect the meningioma. When bleeding from the CS occurred, as might be expected, fibrin glue was injected into the opening of the sinus (Figure 2A). Surgery proceeded in the usual manner (Figure 2B). When the wound was cleaned and inspected, the ICA appeared dusky and darkened (Figure 2C). ICG videoangiography demonstrated a lack of flow in the ICA. Motor-evoked potentials for the ipsilateral hemisphere were also lost. The motor-evoked potential loss and arterial change were unexplained, and thus the patient underwent emergent angiography (Figure 2D). Multiple attempts were made at thrombectomy, using aspiration and a stent retriever, but to no avail. Ultimately, the patient underwent decompressive hemicraniectomy and resection of ischemic tissue. She then experienced a devastating ICA territory stroke (Figure 2E). She was discharged with a tracheostomy and a gastrostomy tube to a long-term acute care facility, where she died several months later.

## Discussion

Hemostasis during CS exposure and anterior clinoidectomy is crucial to maintaining a clear surgical field and performing anatomical dissection safely (10, 11, 13). Direct injection of fibrin glue is one of several techniques used to achieve hemostasis during CS exposure, and it has been demonstrated to be quick, simple, effective, and safe (6, 7, 10–13). Nonetheless, as illustrated by these 2 cases of ICA thrombosis after fibrin glue injection to achieve CS hemostasis, this technique does carry the risk of complications connoting a poor prognosis.

### Effects of Fibrin Glue Injection

Fibrin glue is a solution of concentrated human fibrinogen, bovine thrombin, and calcium chloride delivered with a dual-syringe system that mixes these components as they are injected (15). As the components are combined, the thrombin catalyzes the conversion of fibrinogen to fibrin, inducing the production of a fibrin clot, which then adheres to tissues and forms a hemostatic cast. In neurosurgery, fibrin glue is used mainly to reinforce wound closures, prevent and treat cerebrospinal fluid leakage, and achieve hemostasis along the skull base (6, 9–11, 13, 16–19). When fibrin glue is applied within the CS, it expands to form a local coagulum that temporarily obliterates the main venous channels inside the CS, producing the desired hemostasis without extending to other important neurovascular structures.

### Fibrin Glue Application

The recommended technique for injecting fibrin glue is administering it at 2 points within the CS during anterior clinoidectomy. The first point is located between the V1 and V2 divisions of the trigeminal nerve (during exposure of the whole CS) to block venous inflow to the CS from the petrosal and ophthalmic systems. The second point is posterior to the clinoidal segment of the ICA in the roof of the CS (during anterior clinoidectomy) to block venous inflow from the intercavernous sinuses and the basilar plexus (10). One concern of fibrin glue injection in the CS is that it could result in CS thrombosis syndrome. However, this clinical scenario has not been reported, perhaps because of the usually temporary nature of thrombosis and the redirection of venous flow through other channels.

### Adverse Effects

Fibrin glue injection in the CS is considered a safe technique for hemostasis that has few associated comorbidities (6, 10–13). After it is applied, fibrin glue typically remains within the venous channels where it causes local compression; however, it can affect neural structures in some cases. In 2015, Tavanaiepour et al. (12) reported the first documented case of cranial nerve deficit after an injection of fibrin glue into the CS. In that case, the patient experienced transient trigeminal (V1 and V2 divisions) hypoesthesia that lasted ~18 months. The nerve deficit was attributed to local compression caused by the expansion of the fibrin clot, mimicking balloon compression for trigeminal neuralgia. In animal models, direct injection of fibrin glue in nervous tissue has not demonstrated any caustic or chemical side effects (20, 21).

In 2017, Toyooka et al. (13) reported that direct injection of fibrin glue in the CS altered venous flow in up to 25% of patients, without brain swelling or venous infarction complications. However, the altered venous flow returned to normal after 2–3 months.

To the best of our knowledge, there are no published reports of ICA thrombosis after fibrin glue injection in the CS for hemostasis. After fibrin glue injection, possible causes of ICA thrombosis include direct injection into the ICA through an inadvertent puncture, an abnormal connection (e.g., a cavernous arteriovenous fistula), local compression of the ICA, or an idiosyncratic allergic reaction to the glue. Inadvertent ICA puncture would likely become quickly apparent because of subsequent brisk arterial bleeding, which was not encountered in our 2 patients. Local compression would manifest as ICA stenosis in the cavernous carotid segment on postoperative angiography, which was also not observed in either case. An allergic reaction or other explanation cannot be ruled out in these 2 cases.

### Management

ICG videoangiography is a simple technique that provides real-time information about the patency of vessels (19). As shown in these 2 cases, ICG demonstrated the patency of the right ICA and posterior communicating artery after clinoidectomy and fibrin glue injection in the CS. However, emergency imaging demonstrated complete occlusion of the ICA 2 h after surgery in case 1 and at the end of the tumor resection in case 2. In the setting of an aneurysm clipping, this occlusion might be caused by clip placement, and consideration should be given to adjusting the clips. However, DSA showed no evidence of stenosis caused by the clip. ICA thrombosis occurred progressively in both patients, possibly because of hypercoagulability or an idiosyncratic reaction to fibrin glue that led to thrombus formation during the surgical procedure.

CT and CTA should be conducted immediately when new neurological deficits develop after an injection of fibrin glue in the CS and manipulation of the ICA. DSA and thrombectomy should follow immediately, based on CT findings. Successful reopening of the occluded ICA may not require additional intervention, but infarction and cerebral edema may require decompressive hemicraniectomy. In patients with no evidence of advanced infarction, emergent revascularization may be indicated with an STA-MCA (superficial temporal artery to middle cerebral artery) bypass or an ECA-RAG-M2 MCA (external carotid artery to radial artery graft to M2 to middle cerebral artery) interpositional bypass, depending on collateral circulation and flow demands of the individual patient.

## Limitations

The main limitations of this study are its retrospective nature and presentation as a case report. This study lacks a control group that could be used to establish a causal relationship between fibrin glue injection and thrombosis of the ICA. However, to our knowledge, this is the first report of this complication and should create awareness among physicians who perform this procedure.

## Conclusion

Direct injection of fibrin glue into the CS for hemostasis is an effective technique for controlling venous bleeding and facilitating complex dissection; however, it can be associated with ICA thrombosis and serious ischemia. Although this complication appears to be rare, its recognition should temper the routine use of fibrin glue during anterior clinoidectomy and transcavernous approaches. Patients who experience this complication require emergent intervention to reopen the ICA, revascularize the MCA territory, and decompress brain infarction.

## Data Availability Statement

The original contributions presented in the study are included in the article. Additional inquiries can be directed to the corresponding authors.

## Author Contributions

HA-B and ZC: conception and design. ML and JH: supervision. All authors: data collection, manuscript drafting, critically revised manuscript, and approved final version.

## Conflict of Interest

The authors declare that the research was conducted in the absence of any commercial or financial relationships that could be construed as a potential conflict of interest.

## Publisher's Note

All claims expressed in this article are solely those of the authors and do not necessarily represent those of their affiliated organizations, or those of the publisher, the editors and the reviewers. Any product that may be evaluated in this article, or claim that may be made by its manufacturer, is not guaranteed or endorsed by the publisher.

## Abbreviations

CS: cavernous sinus
CT: computed tomography
DSA: digital subtraction angiography
ECA-RAG-M2 MCA: external carotid artery to radial artery graft to M2 to middle cerebral artery
ICA: internal carotid artery
ICG: indocyanine green
MRA: magnetic resonance angiography
STA-MCA: superficial temporal artery to middle cerebral artery.
